# Only watching others making their experiences is insufficient to enhance adult neurogenesis and water maze performance in mice

**DOI:** 10.1038/srep14141

**Published:** 2015-09-15

**Authors:** Deetje Iggena, Charlotte Klein, Alexander Garthe, York Winter, Gerd Kempermann, Barbara Steiner

**Affiliations:** 1 Department of Neurology, Charité Universitätsmedizin, Charitéplatz 1, 10117 Berlin; 2 German Center for Neurodegenerative Diseases (DZNE) Dresden, Arnoldstraße 18, 01307 Dresden; 3 CRTD – Center for Regenerative Therapies Dresden, Technische Universität Dresden, Fetscherstraße 105, 01307 Dresden; 4 Institute of Biology and Berlin Mouse Clinic for Neurology and Psychiatry, Humboldt-Universität Berlin, Dorotheenstraße 96, 10117 Berlin.

## Abstract

In the context of television consumption and its opportunity costs the question arises how far experiencing mere representations of the outer world would have the same neural and cognitive consequences than actively interacting with that environment. Here we demonstrate that physical interaction and direct exposition are essential for the beneficial effects of environmental enrichment. In our experiment, the mice living in a simple standard cage placed in the centre of a large enriched environment only indirectly experiencing the stimulus-rich surroundings (IND) did not display increased adult hippocampal neurogenesis. In contrast, the mice living in and directly experiencing the surrounding enriched environment (DIR) and mice living in a similar enriched cage containing an uninhabited inner cage (ENR) showed enhanced neurogenesis compared to mice in control conditions (CTR). Similarly, the beneficial effects of environmental enrichment on learning performance in the Morris Water maze depended on the direct interaction of the individual with the enrichment. In contrast, indirectly experiencing a stimulus-rich environment failed to improve memory functions indicating that direct interaction and activity within the stimulus-rich environment are necessary to induce structural and functional changes in the hippocampus.

The exposure to environmental enrichment is beneficial for structural and functional changes in the brain. Living in an enriched environment enhances the survival of newborn neurons in the hippocampus of adult mice, whereas physical activity predominantly stimulates the proliferation of hippocampal precursor cells^1^,^2^. Both external stimuli are additive and thus lead to a remarkable net increase in adult neurogenesis^3^. In several studies, this increased amount of newborn neurons has been linked to the improvement of certain hippocampal-dependent functions including spatial learning^1^,^3^,^4^,^5^,^6^. These observations relate to the medical observation that physical and cognitive activity reduce the risk of memory decline and neurodegenerative disorders^7^,^8^.

As “activity” promotes neurogenesis, motility in a stimulus-rich world might be a strong modulator of neurogenesis-related function. Indeed, in a longitudinal study individual levels of active exploration and territorial coverage (“roaming entropy”) correlated with adult hippocampal neurogenesis^9^. However, an enriched environment is more than merely an incentive for increased levels of motility. Instead it represents a complex inanimate and social stimulation consisting of multiple factors in numerous domains^10^. Due to the complexity of an enriched environment the extent to which individual identifiable factors, including cognitive stimuli, contribute to the positive overall outcome has remained largely unknown.

Usually, mice living in an enriched environment are able to directly interact with their stimulating surrounding^1^,^3^,^6^,^10^. However, considering that a sedentary lifestyle is increasingly common we were in particular interested in the effects of indirect exposure and passive confrontation with such an environment^11^. We asked whether active participation is required for the beneficial effects of environmental enrichment on the brain or whether the merely indirect exposure to sights, sounds and odors of other mice directly experiencing that environment would be sufficient to enhance adult hippocampal neurogenesis. To answer this question we randomly assigned our mice to four different housing conditions (Fig. 1A) and exposed them either directly or indirectly to environmental enrichment for four or eight weeks (Fig. 1B). We conducted histological studies to investigate adult hippocampal neurogenesis and tested the mice in the Morris water maze to assess spatial memory as example of potential functional consequences.

## Results

### Direct interaction with environmental enrichment increases the survival of newborn neurons

Adult neurogenesis was assessed by the standard methodology based on bromodeoxyuridine (BrdU)-incorporation into the dividing precursor cells and immunohistochemical analysis of their progeny^12^. Typically for the enrichment paradigm, direct exposure to the environmental enrichment elicited a strong pro-survival effect on newborn cells four weeks after BrdU-incorporation (Fig. 2A,B; F_3,25_ = 13.809, P < 0.001; post-hoc: ENR versus CTR, P < 0.001; DIR versus CTR, P = 0.001). However, this beneficial effect on neurogenesis was absent in the IND group with prohibited direct interaction and only indirect exposure to an enriched environment (IND versus CTR, P = 0.227). We further explored whether the observed difference was due to an increased number of newborn neurons by estimating BrdU^+^/NeuN^+^ colabeling. ENR and DIR showed significantly more NeuN-positive neurons than CTR (Fig. 2C; F_3,25_ = 11.673, P < 0.0001; post-hoc: ENR versus CTR, P < 0.001; DIR versus CTR; P = 0.003) while the indirect exposure did not affect neuronal differentiation (IND versus CTR, P = 0.401) suggesting that indirect exposure is an inadequate stimulus for adult neurogenesis. Instead, direct interaction is required to enhance the survival of newborn neurons.

### Hippocampal cell proliferation remains unaffected by environmental enrichment

We next examined hippocampal proliferation in transgenic Nestin-GFP mice using BrdU-immunohistochemistry and determined the different subpopulations of neuronal precursor cells by colabeling for neuronal markers^13^,^14^. As predicted, neither direct nor indirect exposure to an enriched environment increased hippocampal cell proliferation 24 hours after BrdU-incorporation (Fig. 2E; F_3,22_ = 0.391; P = 0.761). Similarly, our additional investigations revealed no differences in the subtypes of neuronal precursor cells (Fig. 2F). The groups neither differed in the number of type 1, Nestin^+^/GFAP^+^-cells, (F_3,17_ = 0.730, P = 0.548) type 2a, Nestin^+^-cells, (F_3,21_ = 0.467, P = 0.709) type 2b, Nestin^+^/Dcx^+^-cells (F_3,21_ = 0.704, P = 0.560) nor type 3, Dcx^+^-cells, (F_3,21_ = 0.271, P = 0.846) cells. The histological analysis indicates that the beneficial effects of direct interaction with an enriched environment are in particular due to survival-promoting effects instead of increasing the cell proliferation rate or influencing the distribution of the different subpopulations of neuronal precursor cells.

### Direct interaction with environmental enrichment improves Morris water maze performance

We next related our morphological results to behavioral data. Our mice were tested in a modified reversal learning version of the Morris water maze task that is able to detect alterations in cognitive performance associated with adult neurogenesis^15^. All groups started from the same base line showing no differences regarding the length of swim path during the first trial of day 1 (F_3,37_ = 0.603, P = 0.617) and performed similar over the entire first day. As indicated by the number of crossings at the previous goal location during the first trial after moving the platform to another quadrant all groups learned the task well (Fig. 3A; F_3,37_ = 0.216, P = 0.885). Additionally, no differences were found for swim path lengths during the first trial following goal reversal (day 4, F_3,37_ = 0.599, P = 0.620).

Environmental enrichment had the anticipated beneficial effect on task performance compared to CTR. Post-hoc analysis over all trials revealed that the average swim path to the hidden platform was significantly shorter in ENR (Fig. 3B; Repeated measures ANOVA: F_3,241_ = 10.343, P < 0.001; post-hoc: ENR versus CTR, P = 0.030). This effect appeared to be particular strong at the end of the acquisition phase (day 3, F_3,242_ = 6.920, P < 0.001; post-hoc: ENR versus CTR, P = 0.031). Interestingly, although DIR experienced the same length of exposure to environmental enrichment as mice from the ENR group, the swim path to target shown by DIR mice did not differ from CTR (over all days: DIR versus CTR, P = 0.999) nor on any single day (Day 1: P = 0.135; day 2: P = 0.154; day 3: P = 0.961; day 4: P = 0.971; day 5: P = 1.000). On the second day, DIR even appeared to perform worse than ENR (F_3,242_ = 6.248, P < 0.001 post-hoc: ENR versus DIR, P = 0.050).

Even more surprising was the observation that IND performed worse than CTR in the water maze. Over all trials, IND showed significantly longer swim paths to target (IND versus CTR, P = 0.036). This discrepancy became particularly apparent on day 2 (IND versus CTR, P = 0.012), while on day 4 at least a trend to difference was observable (F_3,242_ = 5.078, P = 0.002; IND versus CTR, P = 0.076). These results suggest that the merely indirect exposure to environmental enrichment is insufficient to improve spatial memory and might even induce certain adverse effects on memory formation.

In addition to this overall analysis of water maze performance, we assessed the effect of different types of enrichment on distinct learning phases. We analyzed the swim path to target separately during acquisition (days 1 to 3) and reversal (days 4 and 5). Remarkably, the inferiority of IND compared to CTR was predominantly present during the reversal phase. While the analysis revealed a difference between IND and CTR on days 4 and 5 (Repeated measures ANOVA: F_3,242_ = 7.549, P < 0.0001; post-hoc: IND versus CTR, P = 0.032), no difference between IND and CTR during acquisition could be detected (Repeated measures ANOVA: F_3,241_ = 6.397, P < 0.001; post-hoc: IND versus CTR, P = 0.379). These results indicate that in IND memory is especially affected in terms of flexibility and the ability to relearn. A similar pattern was revealed comparing DIR to ENR: during acquisition DIR and ENR performed similarly (P = 0.730), but reversal analysis revealed a trend to worse performance in DIR mice, which however remained statistically insignificant (P = 0.087). Neither during acquisition nor during reversal DIR differed from CTR (P = 0.881 and P = 0.987, respectively). The “normal” enrichment group ENR displayed a trend to differ from CTR during acquisition (P = 0.084), while there was no evidence of a difference between ENR and CTR during reversal (P = 0.349). It seems that while ENR possibly outperformed CTR during acquisition, IND and DIR were unable to do likewise and even performed worse during reversal compared to CTR and ENR, respectively. Flexibility seems to be the main aspect of memory formation to be affected by the experimental design of indirect versus direct exposure to environmental enrichment, thereby contributing to the reduced overall performance of IND and DIR in the Morris water maze (Fig. 4).

### Direct interaction with environmental enrichment enhances the quality of spatial learning

We next analyzed qualitative aspects of the spatial learning. First, we plotted the presence probability of the mice in the circular water maze arena. On day 4, the heatmap visualizes the faster reversal learning in ENR. ENR evolved a clear local preference for the new target already during the third trial, whereas the other groups struggled to develop a new preference. Even in DIR a new strong local preference for the new platform location did not evolve as quickly as in ENR. This fits the observation that DIR required a longer swim path to the target than ENR. However, group differences regarding the average distance to target could not be revealed, but between IND and ENR during the third trial on day 4 ((F_3,37_ = 4.786, P = 0.006, IND versus ENR, P = 0.004). Second, we investigated the efficiency of the chosen search strategies and whether these strategies were hippocampus-dependent. To classify the search strategies the sequence of xy-coordinates was analysed using an algorithm introduced previously^15^. The statistical analysis of independence revealed that IND mice more often used hippocampus independent non-spatial like thigmotaxis, random search, scanning and chaining and thus chose less efficient strategies to navigate to the platform than compared to control and the other groups would have been expected (Fig. 3D; chi-square (3) = 34.276, P < 0.001, IND (53.8%) versus CTR (43.9%), DIR (42.1%), ENR (35.4%)). This result adds qualitative details to the observed prolonged latency and swim paths to the platform and indicates that functional plasticity is reduced in IND compared to CTR. With respect to “perseverance”, characterized by an incorrect, prolonged preference for the previous goal on day 4, ENR persevered less than CTR and IND (chi-square (21) = 44.782, P = 0.002, IND (31.3%), CTR (28.3%), DIR (15.3%), ENR (6.7%). This is matching the impression that can be gained from the heatmaps. The general preference of other strategies than perseverance probably contributes to the overall superior performance of ENR (Fig. 4).

## Discussion

Our data indicate that the beneficial effects of environmental enrichment depend on the direct interaction of the individual with that environment. In contrast, merely watching (as well as hearing and smelling) other mice directly experiencing that stimulus-rich environment was insufficient to enhance adult hippocampal neurogenesis and failed to improve memory functions. The prevented direct interaction of the mice with environmental enrichment and other mice close-by even seems to have certain adverse effects on spatial learning.

Generally, our study confirms that environmental enrichment is a strong extrinsic neurogenic stimulus. But we also found the three groups experiencing enrichment in the present experiment (DIR, IND and ENR) to be benefitting differently from the external stimulation. It appeared, for example, that the beneficial effects of an enriched environment on spatial memory were less pronounced in the DIR group than in the classical ENR group. These data were surprising at first. Potential confounders have to be discussed, which would result in a reduced enhancement of spatial memory by environmental enrichment that otherwise is known to be a robust stimulus of learning and memory performance and adult neurogenesis. However, both DIR and ENR groups had indeed the exact same floor size and cage design with the same possibility for physical activity and inter-individual stimulation, but ENR lacked mice in the inner cage. Possibly, the mice living in the inner cage acted as a distracting factor for DIR and thus reduced the positive impact of environmental enrichment on water maze performance. This might likewise apply to IND, which showed the poorest performance in the Morris water maze task. Also here the cage size and design were exactly the same as in CTR. The surroundings of the cages and the experimenters and animal care takers did not change over the experiment period to avoid the potential influence of an additional stimulation or stress. It might be that the proximity of other mice, which are only separated from the own group by a transparent wall, influenced hippocampus-dependent cognitive functions. Potentially, this effect was provoked by the inability of the two groups to directly interact with each other. This observation raises interesting questions about the role of social interactions in the effects elicited by environmental enrichment^16^.

A study from the 1970s is in line with our findings, reporting that rats observing other rats in environmental enrichment neither displayed increased brain weight nor improved exploratory behavior^17^. Similarly, passive motion in a visually stimulating environment did not enhance performance in visual-spatial discrimination tasks^18^. Neither adult hippocampal neurogenesis nor spatial memory have been investigated in these paradigms.

Another study has confronted mice with a locked running-wheel, which, surprisingly, resulted in an increase in cell proliferation in the hippocampus^19^. Even though in that study the running-wheel could not be used for running, the mice could still directly interact with the running-wheel which thus by itself might be sufficient to contribute to enrichment, resulting in the observed effect on adult neurogenesis. We did not see a similar effect on hippocampal cell proliferation in any of our groups though, which is consistent with the prevailing notion that enriched environments primarily affect cell survival rather than precursor cell proliferation.

That previous evidence is also relevant with respect to the obvious notion that the levels of physical activity will have differed between our groups. Given that as yet no monitoring system is available that would allow measuring the low levels of motility in our cage-in-cage set up we do not know to what exact degree our groups differed in this respect. However, in none of the groups in the larger enriched cages did we observe increased precursor cell proliferation as it is normally displayed by mice with access to a running wheel^2^. In fact, it was the expected survival-promoting effect on newborn neurons that resulted in increased levels of adult neurogenesis in ENR and DIR.

Physical inactivity and the mere indirect exposure to external stimulation are characteristic of a sedentary lifestyle as it is found in modern humans. Such sedentary lifestyle is characterized by sitting in conjunction with excessive television consumption and computer use and is associated with increased morbidity and mortality^20^,^21^,^22^,^23^. Even though a direct transfer of our results to a human lifestyle is not advisable, cautious extrapolation to the human condition can be instructive since inactivity strongly interferes with mental and physical health^20^,^21^,^22^,^23^.

While the “couch-potato analogy” is tempting and suggestive, a cautionary note is nevertheless required: we have been studying a highly reductionistic situation in mice. Lifestyle is a far more complex event consisting of multiple contributing factors. However, investigations in inbred mice with constant genetic factors enable scientists to assess the exclusive impact of emblematic environmental constellations in laboratory animals thus providing the opportunity to deepen our understanding of extrinsic neurogenic stimulation.

At present, few good experimental models are available to elucidate the complexity of lifestyle compounds and lifestyle interventions^24^. Some researcher consider housing in a standard cage as model for inactivity, while other researchers have introduced an audio-visual overstimulation paradigm for infant mice resembling a television-like set-up and revealed ensuing deficits in cognitive performance^25^,^26^.

Despite all limitations and potential confounders, our findings are first experimental steps in addressing the biological basis of the more extensive multifactorial situation in humans. In the future, advanced lifestyle experiments are needed to assess the consequences of unhealthy lifestyles. In any case, our results do support the clear indications that physical activity and direct interaction with the environment is superior to second-hand experience.

## Materials and Methods

### Animals

A total number of 97 female mice at the age of six to seven weeks were assigned to our experiment. 71 C57Bl/6N mice were purchased from Charles River for the assessment of cell survival and spatial memory while 26 transgenic Nestin-GFP mice, expressing green fluorescent protein under the promotor of nestin on a C57Bl/6N background, were obtained from FEM Beyer to enable the qualification of proliferating neuronal precursor cells^13^. All experimental procedures were conducted according to federal laws, and approved by the appropriate local authorities (LAGESO Berlin, Germany).

### Experimental set-up

**Figure 1:**
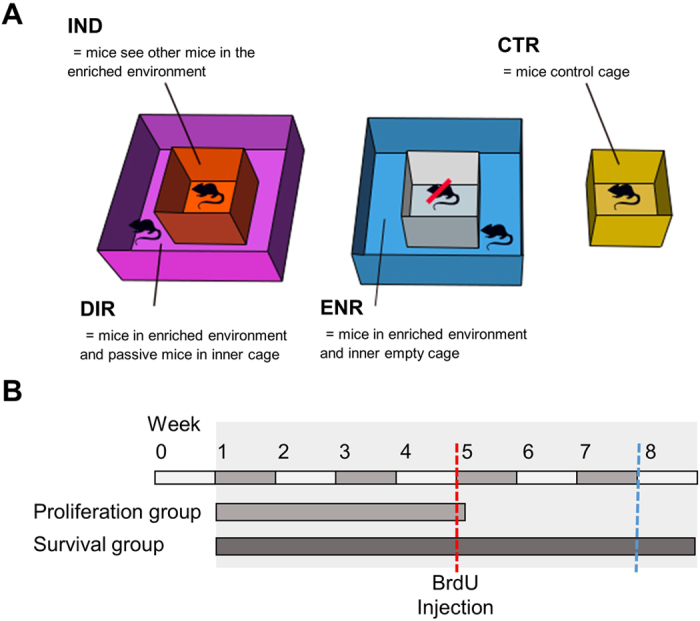
Experimental set-up. (**A**) Housing conditions. Mice lived in and directly experienced an enriched environment (DIR) while the mice in the inner standard cage indirectly experienced the surrounding enriched environment and its inhabitants (IND). Mice lived in an enriched environment which contained an uninhabited inner cage (ENR). Mice lived in a standard cage without any confrontation to environmental enrichment (CTR). The measures of the ENR/DIR cage: 0.74 m × 0.3 m × 0.74 m (W/H/D), the measures of the CTR/IND cage: 0.27 m × 0.15 m × 0.42 m (W/H/D). (**B**) Experimental timeline. Mice received three BrdU-injections on day 28 of the experiment. To assess cell proliferation mice were killed 24h after injection, to investigate cell survival mice were killed four weeks after injection. Spatial memory was assessed during the eighth week of the experiment.

The mice were randomly assigned to four different groups with a minimum of n = 5. After an adaption period, mice were placed into their assigned housing condition (Fig. 1A). The first group (IND) was housed in a simple standard cage (0.27 m × 0.15 m × 0.42 m) placed in the centre of a large enriched environment cage (0.74 m × 0.3 m × 0.74 m) and thus enabled to indirectly experience the stimulus-rich surrounding. The second group (DIR) was housed in the former mentioned enriched environment, enabled to directly experience and to physically interact with this stimulus-rich surrounding. IND and DIR were permanently confronted with each other and only separated by an acrylic Perspex wall and grids. The third group (ENR) was housed in an enriched environment cage (0.74m × 0.3m × 0.74m) similar to the one of the DIR group, but containing an uninhabited inner standard cage. The fourth group (CTR) was housed in a standard cage (0.27 m × 0.15 m × 0.42 m) without any confrontation to environmental enrichment or any other mice and held under control conditions. Environmental enrichment was rearranged two to three times a week and consisted of a rearrangeable set of plastic tubes, cardboard tubes, grids, colourful cartons, varying shelters, and changing food and drinking places. All mice received food and water ad libitum and remained in a constant light/dark cycle of 12 hours. The standard cage housed 5 to 7 mice at the same time, while the ENR/DIR cage housed 5 to 10 mice at the same time. A total of 22 mice were used as CTR, four standard cages were used for this condition. A total of 23 mice were used as IND, four standard cages were used for this condition. A total of 22 mice were used as ENR, three ENR cages were used for this condition. A total of 29 mice were used as DIR, four DIR cages were used for this condition. A total of 26 transgenic GFP-nestin mice (CTR: N = 5, IND: N = 6, ENR: N = 5, DIR = 10) spent four weeks in the experiment and were used to analyse the proliferation of dividing precursor cells in the hippocampus. A total of 29 C57Bl/6 mice (CTR: N = 7, IND: N = 6, ENR: N = 7, DIR: N = 9) spent eight weeks in the experiment to assess the survival of newborn cells. A total of 41 C57Bl/6 mice were tested in the Morris water maze (CTR: N = 10, IND: N = 11, ENR: N = 10, DIR: N = 10) and visuo-spatial memory was assessed (Fig. 1B). The equipment of the room, experimentators and animal facility care personel remained stable over the whole experimental period.

### BrdU-injection protocol

To determine the proliferation and survival of newborn cells, mice were injected with 5-Bromo-2′-deoxyuridine (BrdU, Sigma) dissolved in 0.9% saline. BrdU is a thymidine analogon which incorporates into the DNA of dividing precursor cells and thus enables to assess proliferating cells. The mice received three BrdU-injections (50mg/kg) intraperitoneally within twelve hours on day 28 of the experiment. The Nestin-GFP mice were transcardially perfused 24 hours after the injection in order to determine the proliferation rate of cells while the other mice were perfused four weeks after BrdU-injection to assess the survival of the newborn cells (Fig. 1B).

### Histological tissue preparation

All mice were deeply anaesthesized by an overdose of Ketamine and killed by transcardially perfusion with cold 0.1M phosphate-buffer saline (PBS, pH 7.4) followed by 4% paraformaldehyde. The mice were decapitated, the brains dissected, and stored for 24 hours in 4% paraformaldehyde for postfixation. After postfixation, the brains were dehydrated in 30% sucrose until they sunk to the bottom. Subsequently, the brains were frozen in liquid nitrogene and stored at –80°C till further processing. For histological analysis, the brains were cut into 40μm thick coronal sections using a cryostat (Leica DM 1850) and stored in cryprotectant at 4°C till staining.

### Immunohistochemistry and analysis

To quantify the number of newborn cells, one-in-a-6 series (240 μm apart) of the sections were stained for BrdU-detection as described previously^12^. Sections were pretreated with 0.6% H_2_O_2_ for 30 minutes to block endogenous tissue peroxidase. After washing the free-floating sections in PBS, DNA was denaturated with 2N HCL for 30 minutes at 37 °C. The sections were then rinsed in borate buffer for 10 minutes followed by washing with PBS. To prevent unspecific antibody binding, sections were treated with PBS+ (0.1% TritonX-100, 3% donkey serum) for 30 minutes before being incubated overnight with the primary antibody against BrdU (rat, Biozol) diluted 1:500 in PBS+. The primary antibody was washed out with PBS the next day. Subsequently, the sections were blocked again with PBS+ for 25 minutes followed by incubation with the biotinylated secondary antibody (anti-rat, Dianova) diluted 1:250 in PBS+ for two hours at room temperature. After washing out the secondary antibody, avidin biotin peroxidase complex reagent (ABC Elite, Vector Laboratories) at a concentration of 9μl per 1ml PBS was applied for one hour and washed out. Diaminobenzidine (DAB, Sigma-Aldrich) was used as chromogene at the concentration of 0.025 mg/ml in destilled water and Tris-buffer with 0.01% H_2_O_2_ and 0.04% nickel chloride. After washing in PBS and destilled water, the free floating sections were mounted onto superfrost glass slides (Menzel), dehydrated in ascending concentrations of ethanol, cleared in ProTaqs®Clear and coverslipped with ProTaqs®PARAmount. The same blinded researcher analysed DAB-stained sections for BrdU-positive cells throughout the rostrocaudal extent of the granule cell layer in both hippocampi except for the uppermost focal layer. The resulting number was multiplied by six to obtain an estimation of the total number of BrdU-positive cells in the dentate gyrus. Exemplary pictures of BrdU-positive cells were taken by differential interference contrast (DIC)-microscopy (Olympus BX50). Unessential parts of the pictures were removed, but no further manipulation occurred.

### Immunofluorescence staining and analysis

To determine the phenotype of the newborn cells, one-in-a-12 series (480μm apart) of the sections were double- or triple-labelled by the proliferating marker BrdU, the marker for mature neurons NeuN or marker for premature neurons Doublecortin, GFP for nestin-staining, and GFAP. After DNA denaturation, rinsing in borate buffer and blocking of unspecific antibody binding as described above, the sections were incubated overnight with the primary antibody diluted in PBS+ in the following concentrations: anti-BrdU 1:500 (rat, AbD Serotec), anti-NeuN 1:100 (mouse, Millipore), anti-GFP 1:250 (rabbit, Abcam), anti-Doublecortin 1:200 (goat, Santa-Cruz), anti-GFAP 1:200 (goat, Santa-Cruz). The next day, sections were rinsed followed by blocking with PBS+, and incubated with the secondary antibodies diluted in PBS+ at room temperature for four hours. The following secondary fluorochrome antibodies and concentrations were used: anti-rat Rhodamine X, 1:250 (Dianova), anti-mouse Alexa 488, 1:1000 (Invitrogen), anti-rabbit Alexa 488, 1:1000 (Invitrogen), anti-goat Alexa 647, 1:100 (Invitrogen). After incubation, sections were washed, mounted, dehydrated, cleared, and coverslipped. Immunofluorescence stained sections were analysed by taking confocal z-stacks scanned at 1μm intervals using a Leica TCS SP2. Fifty randomly selected BrdU-positive cells within the granule cell layer were investigated for co-expression of additional neuronal marker. The ratio of the neuronal phenotypes was multiplied by the total number of BrdU-positive cells and yielded an estimation of the absolute numbers of newborn neurons within the granule cell layer.

### Behavioural testing and analysis

To assess spatial memory, a total of forty-one mice (CTR: N = 10, IND: N = 11, ENR: N = 10, DIR: N = 10) were tested in a modified reversal learning version of the Morris water maze as described previously^15^. For five consecutive days, mice were trained to navigate through opaque water to a hidden platform 1 cm below the surface of a circular tank (1.2m diameter). The temperature was kept constant at 19 °C to 20 ° C. Every day consisted of six trials with a maximum duration of 120 seconds and an inter-trial interval time of 30 minutes. The starting position changed daily, but was kept constant during the day (Fig. 3D). The platform was relocated into the opposite quadrant on day four. In case a mouse failed to find the platform, it was guided to the platform and remained there for 15 seconds. Animals were tracked and recorded by Viewer^3^ (Biobserve). The lengths of the swim paths to target and the number of crossings of the previous target zone were automatically analysed by Viewer^3^. To investigate qualitative properties of the learning in the Morris water maze, classification and analysis of search strategies were performed. Therefore, the recorded xy-coordinates were transferred into a Matlab-script (Mathworks, USA) and automatically analysed based on the algorithm elaborated previously^15^. Search strategies were classified as spatial or non-spatial strategies and chi-square-independence test was used to determine if groups performed more or less often spatial or non-spatial strategies as expected. Furthermore, the xy-coordinates were used to depict the spatial preference of the mice in heat maps of presence probability drafted automatically by the Matlab-script.

### Statistical analysis and data presentation

**Figure 2:**
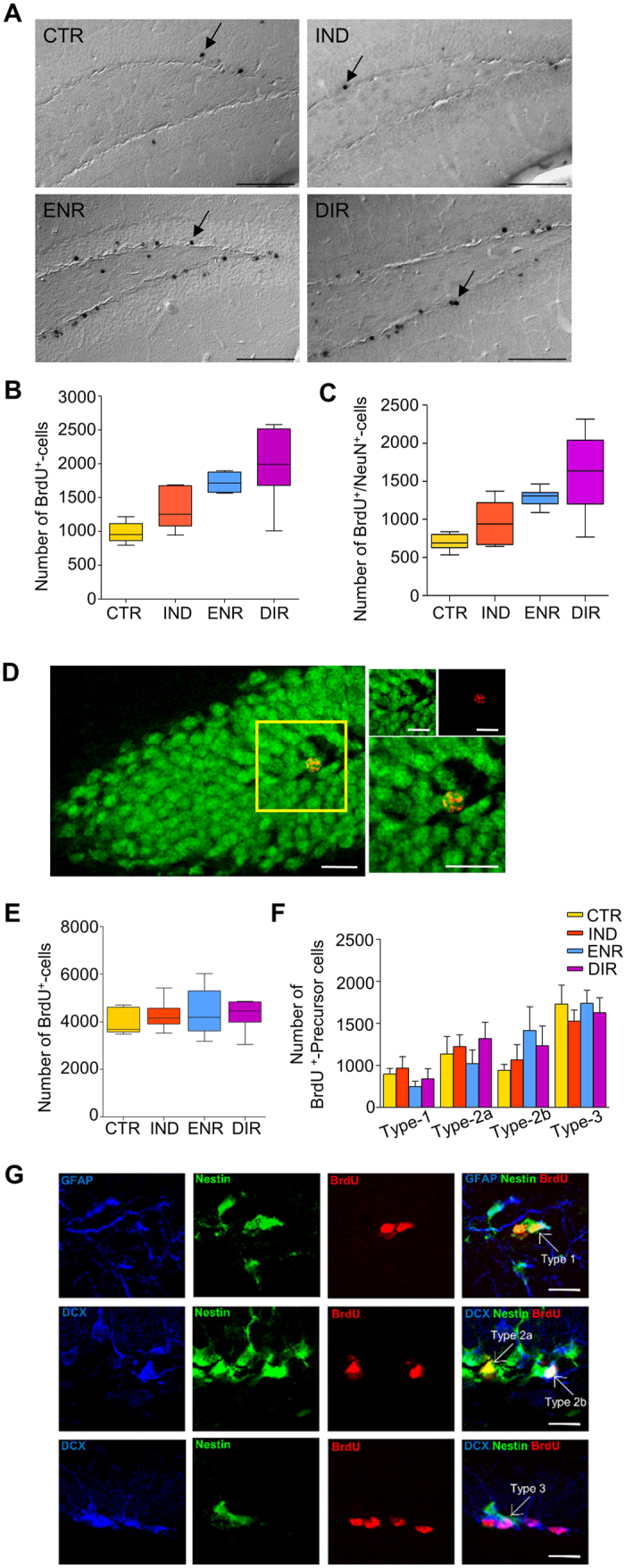
Newborn cells in the granule cell layer of the dentate gyrus. (**A**) Representative DIC-images of BrdU-immunohistochemistry in CTR, IND, ENR and DIR showing four weeks old cells. Arrows highlight exemplary BrdU^+^-cells. Scale bar, 100 μm. (**B**) Absolute number of survived BrdU^+^-cells four weeks after BrdU-incorporation. ENR and DIR displayed significantly more cells (ENR versus CTR, P < 0.001; DIR versus CTR, P = 0.001) while IND and CTR did not differ (IND versus CTR, P 0.227). (**C**) Absolute number of survived neurons colabeled by BrdU and the neuronal marker NeuN. ENR and DIR (ENR versus DIR, P = 0.481) did not differ, but showed increased neurogenesis compared to CTR (ENR versus CTR, P < 0.001; DIR versus CTR, P = 0.003) while IND did not differ from CTR and ENR (IND versus CTR, P = 0.401; IND versus ENR, P = 0.219), but from DIR (IND versus DIR, P = 0.048). CTR, N = 7; IND, N = 6; ENR, N = 7; DIR, N = 9. (**D**) Representative confocal images of NeuN^+^ (green)/BrdU^+^ (red) cells appearing orange when colabeled. Scale bars 20 μm. (**E**) Absolute number of BrdU^+^-cells 24 hours after BrdU-incorporation. Environmental enrichment did not affect cell proliferation rate (P = 0.761). CTR, N = 5; IND, N = 6; ENR, N = 5; DIR, N = 10. (**F**) Absolute number of BrdU^+^-cells in the subpopulations of hippocampal proliferating neurons 24 hours after BrdU-incorporation. Environmental enrichment did not affect any neuronal subpopulation of precursor cells. (**G**) Representative confocal images of BrdU-colabeled type 1 (GFAP^+^/Nestin^+^), type 2a (Nestin^+^), type 2b (Nestin^+^/Dcx^+^) and type 3 (Dcx^+^) neuronal precursor cells. Scale bars 20 μm. Data presented as boxplots with a centre line as median, Tukey-style whiskers extend 1.5 times the interquartile range from 25^th^ and 75^th^ percentiles and dots represent outliers. Numbers of precursor cells shown as mean± s.e.m.

**Figure 3:**
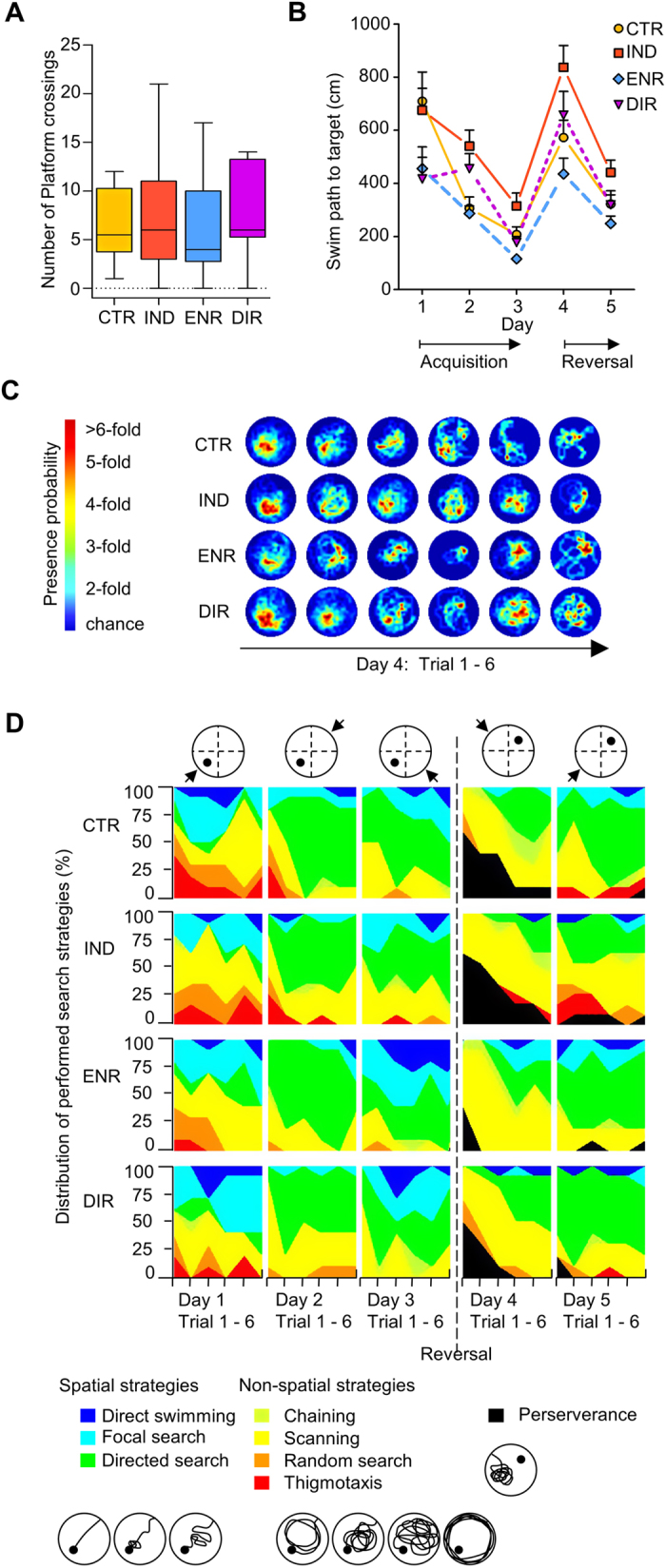
Performance in a reversal-learning type of the Morris water maze. (**A**) All groups learned to navigate to the platform during the acquisition phase (days 1 to 3) thus the groups did not differ in the total number of crossing of the previous target zone during the first trial on day four after the platform was relocated. (P = 0.885). (**B**) The ability to reach the platform as fast as possible differed between the groups. ENR displayed a significantly shorter swim path than CTR while DIR only differed from IND but not from CTR. IND needed even a significantly longer swim path than CTR. (IND versus ENR, P < 0.001; IND versus DIR, P = 0.016; CTR versus ENR, P = 0.030; CTR versus IND, P = 0.036; ENR versus DIR, P = 0.141; CTR versus DIR, P = 0.999) (**C**) Heatmaps depict the development of spatial preferences in the water tank after the platform had been relocated into the opposite quadrant on day four. Although no statistical difference regarding distance to target could be revealed but between IND and ENR in trial 3 (F_3,37_ = 4.786, P = 0.006, IND versus ENR, P = 0.004), the heatmaps visualize ENR’s faster reversal learning and development of a local preference for the new target already during the third trial while the other groups struggled to develop a new preference. (**D**) Distribution of performed search-strategies during the five days. IND used more inefficient non-spatial strategies to reach the goal than expected (Chi-square = 34.276, P < 0.001; IND 58.4%, CTR 43.9%, DIR 42.1%, ENR 35.4%). Number of platform crossings presented as boxplots with a centre line as median, Tukey-style whiskers extend 1.5 times the interquartile range from 25^th^ and 75^th^ percentiles. Swim path to target presented as mean± s.e.m. CTR, N = 10; IND, N = 11; ENR, N = 10; DIR, N = 10.

**Figure 4:**
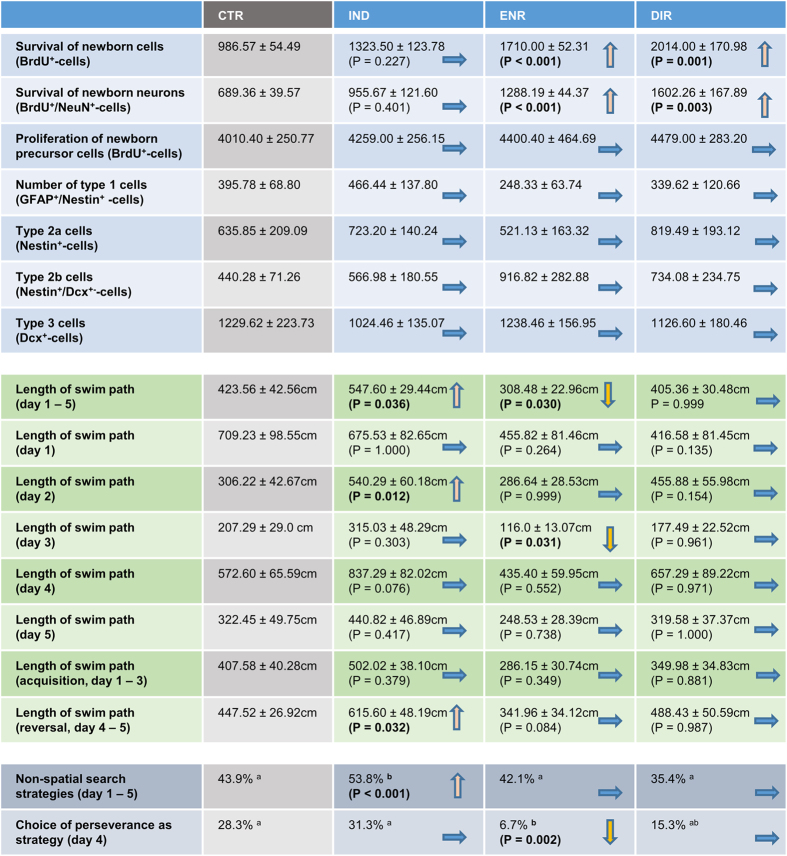
Table of results. Showing an overview of the experimental results regarding histology, length of swim path and choice of search strategies during the Morris water maze. The groups IND, ENR and DIR are compared to CTR. Data is presented as mean value ± s.e.m. In case of significant main effects, the p-value of the followed post-hoc analysis is presented.

Statistical analysis was performed in SPSS 21. The metric variables “number of cells” and “crossing of platform” were analysed with one-way ANOVA while the metric variable “length of swim path” was analysed using repeated measures ANOVA. For comparison between groups, post-hoc tests were used when main analysis revealed significance and Levene’s test was performed to validate homogeneity of variances. Bonferroni-correction was chosen for post-hoc analysis in case of variance homogeneity and Tamhane’s-T2 was selected in case of variance inhomogeneity. To determine an association between housing condition and the nominal variable hippocampal-dependent search strategy respectively hippocampal-independent search strategy the chi-square-independence test was applied. Therefore, the different search strategies were classified as hippocampal-dependent “spatial” strategies or as hippocampal-independent “non-spatial” strategies. According to the literature, the following strategies were defined as “spatial”: “directed search”, “focal search”, “direct swimming” while the following strategies were defined as “non-spatial”: “thigmotaxis”, “random search”, “scanning”, “chaining”^15^,^27^. Likewise, chi-square-test was selected for assessment of “perseverance” and its association with housing conditions. For all applied statistical tests, the level of significance was set to the conventional level of 0.05. Absolute numbers of cells (Fig. 2B,C,E) and absolute numbers of platform crossings (Fig. 3A) are presented as boxplots with a center line as median, Tukey-style whiskers extend 1.5 times the interquartile range from 25^th^ and 75^th^ percentiles. The length of the swim path to reach the hidden platform is presented as mean+ s.e.m (Fig. 3B).

## Additional Information

**How to cite this article**: Iggena, D. *et al*. Only watching others making their experiences is insufficient to enhance adult neurogenesis and water maze performance in mice. *Sci. Rep*. **5**, 14141; doi: 10.1038/srep14141 (2015).

## References

[b1] Kempermann G., Kuhn H. G. & Gage F. H. More hippocampal neurons in adult mice living in an enriched environment. Nature 386, 493–495 (1997). 9087407 10.1038/386493a0

[b2] Van Praag H., Kempermann G. & Gage F. H. Running increases cell proliferation and neurogenesis in the adult mouse dentate gyrus. Nature Neurosci 2, 266–270 (1999). 10195220 10.1038/6368

[b3] Fabel K. . Additive effects of physical exercise and environmental enrichment on adult hippocampal neurogenesis in mice. Front. Neurosci 3, 10.3389/neuro.22.002.2009 (2009). PMC2858601 20582277

[b4] Kempermann G., Kuhn H. G. & Gage F. H. Experience-induced neurogenesis in the senescent dentate gyrus. J. Neuroscience 18, 3206–3212 (1998). 9547229 10.1523/JNEUROSCI.18-09-03206.1998 PMC6792643

[b5] Van Praag H., Christie B. R., Seinowski T. J. & Gage F. H. Running enhances neurogenesis, learning, and long-term potentiation in mice. Proc. Natl. Acad. Sci. 96, 13427–13431 (1999). 10557337 10.1073/pnas.96.23.13427 PMC23964

[b6] Nilsson M., Perfilieva E., Johansson U., Orwar O. & Eriksson P. S. Enriched environment increases neurogenesis in the adult rat dentate gyrus and improves spatial memory. J. Neurobiol 39, 569–578 (1999). 10380078 10.1002/(sici)1097-4695(19990615)39:4<569::aid-neu10>3.0.co;2-f

[b7] Laurin D., Verreault R., Lindsay J., MacPherson K. & Rockwood K. Physical activity and risk of cognitive impairment and dementia in elderly persons. Arch. Neurol 58, 498–504 (2001). 11255456 10.1001/archneur.58.3.498

[b8] Radak Z. . Exercise plays a preventive role against Alzheimer’s disease. J. Alzheimers Dis. 20, 777–783 (2010). 20182027 10.3233/JAD-2010-091531

[b9] Freund J. . Emergence of individuality in genetically identical mice. Science 340, 756–759 (2013). 23661762 10.1126/science.1235294

[b10] Rosenzweig M. R., Bennett E. L., Herbert M. & Morimoto H. Social grouping cannot account for cerebral effects of enriched environment. Brain. Res. 153, 563–576 (1978). 698794 10.1016/0006-8993(78)90340-2

[b11] Atienza A. A. . Identifying sedentary subgroups: The NCI health information national trends survey. Am. J. Prev. Med. 31, 383–390 (2006). 17046409 10.1016/j.amepre.2006.07.024 PMC1934418

[b12] Lesemann A. . MPTP-induced hippocampal effects on serotonin, dopamine, neurotrophins, adult neurogenesis and depression-like behavior are partially influenced by fluoxetine in adult mice. Brain. Res. 1457, 51–69 (2012). 22520437 10.1016/j.brainres.2012.03.046

[b13] Yamaguchi M., Saito H., Suzuki M. & Mori K. Visualization of neurogenesis in the central nervous system using nestin promotor-GFP transgenic mice. Neuroreport 11, 1991–1996 (2000). 10884058 10.1097/00001756-200006260-00037

[b14] Kempermann G., Jessberger S., Steiner B. & Kronenberg G. Milestones of neuronal development in the adult hippocampus. Trends Neurosc. 27, 447–52 (2004). 10.1016/j.tins.2004.05.013 15271491

[b15] Garthe A., Behr J. & Kempermann G. Adult-generated hippocampal neurons allow the flexible use of spatially precise learning strategies. PLoS One 4, e5464, 10.137/journal.pone.0005464 (2009). 19421325 PMC2674212

[b16] Freund J. . Association between exploratory activity and social individuality in genetically identical mice living in the same enriched environment. Neuroscience. 10.1016/j.neuroscience.2015.05.027 (2015). 25987202

[b17] Ferchim P. A. & Bennett E. L. Direct contact with enriched environment is required to alter cerebral weights in rats. J. Comp. Physiol. Psych. 88, 360–367 (1975). 10.1037/h0076175 1120811

[b18] Held R. & Hein A. Movement-produced stimulation in the development of visually guided behaviour. J. Comp. Physiol. Psych. 56, 872–876 (1963). 10.1037/h0040546 14050177

[b19] Bednarczyk M. R. . Distinct stages of adult hippocampal neurogenesis are regulated by running and the running environment. Hippocampus 21, 1334–1347 (2011). 20623741 10.1002/hipo.20831

[b20] Kim Y. . Association between various sedentary behaviours and all-cause, cardiovascular disease and cancer mortality: the Multiethnic Cohort Study. Int. J. Epidemiol. 42, 1040–1056 (2013). 24062293 10.1093/ije/dyt108 PMC3781003

[b21] Wilmot E. G. . Sedentary time in adults and the association with diabetes, cardiovascular disease and death: systematic review and meta-analysis. Diabetologia 55, 2895–2905 (2012). 22890825 10.1007/s00125-012-2677-z

[b22] Sund A. M., Larsson B. & Wichstrøm L. Role of physical and sedentary activities in the development of depressive symptoms in early adolescence. Soc. Psychiatry. Psychiatr. Epidemiol. 46, 431–441 (2011). 20358175 10.1007/s00127-010-0208-0

[b23] Lindstrom H. A. . The relationships between television viewing in midlife and the development of Alzheimer’s disease in a case-control study. Brain and Cognition 58, 157–165 (2005). 15919546 10.1016/j.bandc.2004.09.020

[b24] Bilimoria P. M., Hensch T. K. & Bavelier D. A mouse model for too much TV? Trends Cogn. Sci. 16, 529–531 (2012). 22999015 10.1016/j.tics.2012.09.001 PMC3482269

[b25] Cummins R. A., Livesey P. J. & Evans J. G. A developmental theory of environmental enrichment. Science 197, 692–694 (1977). 877587 10.1126/science.877587

[b26] Christakis D. A., Ramirez J. S. B. & Ramirez J. M. Overstimulation of newborn mice leads to behavioural differences and deficits in cognitive performance. Sci. Rep. 2, 546, 10.1038/srep00546 (2012). 22855702 PMC3409385

[b27] Garthe A., Huang Z., Kaczmarek L., Filipkowski R. K. & Kempermann G. Not all water mazes are created equal: cyclin D2 knockout mice with constitutively suppressed adult hippocampal neurogenesis do show specific spatial learning deficits. Genes, Brain and Behavior 13, 357–364, 10.1111/gbb.12130 (2014). PMC4314690 24602283

